# Fibrinogen-to-albumin ratio: a new biomarker to identify inflammatory bowel disease in active stage

**DOI:** 10.3389/fmed.2025.1460975

**Published:** 2025-02-19

**Authors:** Xiao-Fu Chen, Zhi-Ming Huang, Xie-Lin Huang

**Affiliations:** ^1^Center for General Practice Medicine, Department of Gastroenterology, Zhejiang Provincial People’s Hospital (Affiliated People’s Hospital), Hangzhou Medical College, Hangzhou, China; ^2^Department of Gastroenterology and Hepatology, The First Affiliated Hospital of Wenzhou Medical University, Wenzhou, China

**Keywords:** albumin, biomarker, fibrinogen, inflammatory bowel disease, activity

## Abstract

**Introduction:**

The objective of our study was to externally validate the value of the fibrinogen-to-albumin ratio (FAR), a new biomarker used to identify active inflammatory bowel disease (IBD).

**Materials and methods:**

A total of 245 ulcerative colitis (UC) and 543 Crohn’s disease (CD) patients were included in our study. Multivariate logistic regression analysis was used to investigate the independent association between FAR and disease activity in patients with UC or CD. The area under the receiver operating characteristic curve was used to assess the prediction accuracy of biomarkers in distinguishing disease states.

**Results:**

Multivariate logistic regression analysis identified the FAR as the strongest predictor to discriminate disease activity of UC (odds ratio: 24.871, 95% confidence interval: 9.831–38.912, *p* < 0.001) and CD (odds ratio: 28.966, 95% confidence interval: 21.009–37.250, p < 0.001). The FAR gave the highest area under the curve in identifying both active UC (0.870, 95% confidence interval: 0.824–0.916) and CD (0.925, 95% confidence interval: 0.904–0.946). The probability of both UC and CD patients being in the active stage significantly increased when the FAR was more than or equal to the optimal cutoff values.

**Conclusion:**

The FAR, a simple prognostic indicator, performs well in identifying active IBD.

## Introduction

Inflammatory bowel disease (IBD) is a chronic and recurrent immunological disorder with an increasing incidence and prevalence over time (1–3). The main types of IBD, Crohn’s disease (CD) and ulcerative colitis (UC), are believed to be caused by the dysfunction of the immune system (4). Early detection of IBD activity is crucial for timely treatment and effective prevention of complications, ultimately improving patients’ quality of life and prognosis (5–7). Endoscopic biopsy remains the gold standard for assessing and monitoring inflammatory activity in IBD, while it is an invasive procedure that poses challenges in specimen collection and can be costly.

The neutrophil-to-lymphocyte ratio (NLR), platelet-to-lymphocyte ratio (PLR), and lymphocyte-to-monocyte ratio (LMR) obtained from the complete blood count (CBC) have been demonstrated as effective markers for predicting IBD activity and severity (8–11). Red blood cell distribution width (RDW) has shown good ability in evaluating disease activity, specifically in CD (12). C-reactive protein (CRP) and erythrocyte sedimentation rate (ESR) have been demonstrated as significant biomarkers for early diagnosis of IBD and accurate monitoring of IBD activity (13–15). Inflammation and coagulation are interdependent processes, each activating and propagating the other, which is crucial for the progression of IBD (16, 17). Distinct pro-inflammatory stimuli activate the clotting cascade, which subsequently propagates the inflammatory state by activating signaling pathways or recruiting more inflammatory cells to inflamed tissues (18). Fibrinogen can modify various aspects of inflammatory cell function by engaging leukocytes through different cellular receptors and mechanisms (19). In our previous study, we externally validated for the first time fibrinogen’s diagnostic ability to identify patients with IBD in the active stage and demonstrated that fibrinogen had a high discriminative capacity for active IBD (20). Albumin is a negative acute-phase protein, and its synthetic rate has a direct correlation with the severity of acute inflammation (21). Additionally, albumin was deemed to possess a strong discriminatory ability in identifying children with CD (22). Thus, we proposed a novel biomarker, the fibrinogen-to-albumin ratio (FAR), for identifying active IBD. This study aims to assess the association between FAR and IBD activity and to evaluate the diagnostic ability of FAR in detecting active IBD.

## Materials and methods

### Materials

The research was a retrospective observational study. We included IBD patients admitted to the First Affiliated Hospital of Wenzhou Medical University from September 2011 to September 2019. The diagnosis of IBD was based on clinical evidence such as diarrhea, abnormal laboratory findings, imaging findings, endoscopic findings, and histopathology. Patients meeting the following criteria were excluded: other immune-related diseases, infectious disease, carcinoma, liver cirrhosis, renal or heart or respiratory failure, and loss of fibrinogen or albumin data on admission. We excluded patients with clinical, laboratory, and histological evidence of infectious colitis, radiation colitis, indeterminate colitis, microscopic colitis, or with a doubtful diagnosis of IBD. The clinical data we collected included demographic data, laboratory parameters, and endoscopic findings. The FAR was calculated by dividing fibrinogen by albumin. Disease activity of UC and CD was assessed by the Total Mayo Score and Crohn’s Disease Activity Index (CDAI), respectively. Total Mayo Score was a comprehensive scoring system that contained stool frequency, rectal bleeding, endoscopic findings, and the physician’s global assessment (23, 24). The Total Mayo Score can simply and effectively classify patients with UC, and a score of more than 2 indicated that patients with UC were in the active stage (23, 24). The CDAI criteria included body weight, general health condition, hematocrit, abdominal pain severity, daily blood stool count, and complications (25). The CDAI of more than 150 indicated that patients with CD were in the active stage (25). The ethics committee of the First Affiliated Hospital of Wenzhou Medical University approved this research.

### Statistical methods

The Mann–Whitney *U*-test was used to compare quantitative variables, which were presented as median [interquartile range (IQR)]. The chi-square test or Fisher’s exact test was used to compare categorical variables, which were presented as absolute numbers (frequencies). Multivariate logistic regression analysis was used to investigate the independent association between biomarkers and disease activity in patients with UC or CD to calculate the odds ratio with a confidence interval of 95%. The relationship between FAR and IBD activity was determined by the Spearman’s correlation analysis. The area under the receiver operating characteristic curve (AUROC) was used to assess the prediction accuracy of biomarkers in distinguishing disease states. The AUROCs of various biomarkers were compared using the DeLong test (26). We compared sensitivity, specificity, positive predictive value, negative predictive value, positive likelihood ratio, and negative likelihood ratio at an optimum cutoff value according to the curve. We divided patients in UC and CD cohorts, respectively, into two groups based on the optimum cutoff values for the FAR. All tests were two-sided, and a *p*-value less than 0.05 was considered statistically significant. All statistical procedures were performed with STATA (version 14.0; StataCorp, State of Texas, USA).

## Results

**Figure 1:**
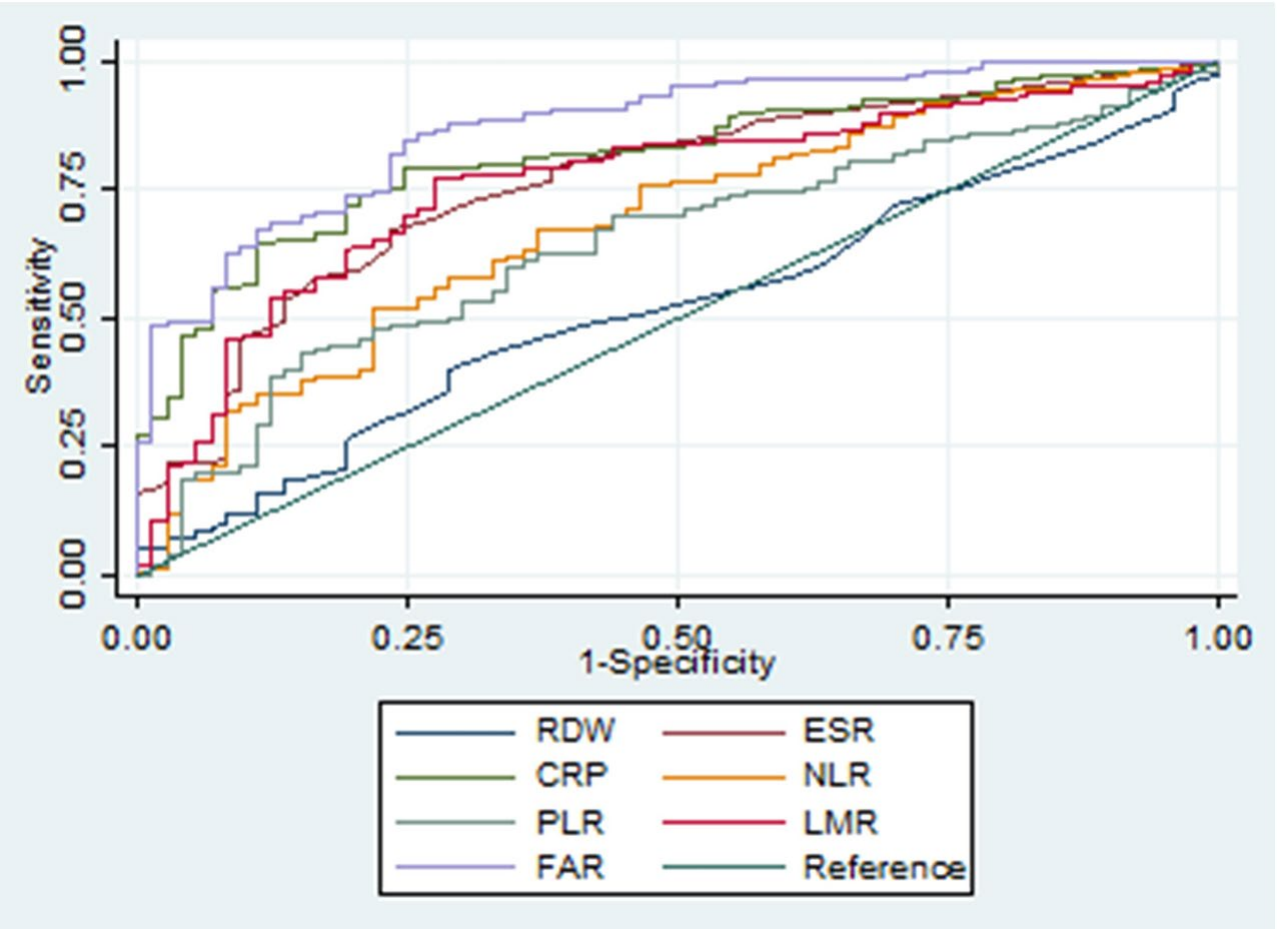
Receiver operating characteristic curves of various biomarkers in identifying active ulcerative colitis. RDW, red cell distribution width; ESR, erythrocyte sedimentation rate; CRP, C-reactive protein; NLR, neutrophil-to-lymphocyte ratio; PLR, platelet-to-lymphocyte ratio; LMR, lymphocyte-to-monocyte ratio; FAR, fibrinogen-to-albumin ratio.

**Figure 2:**
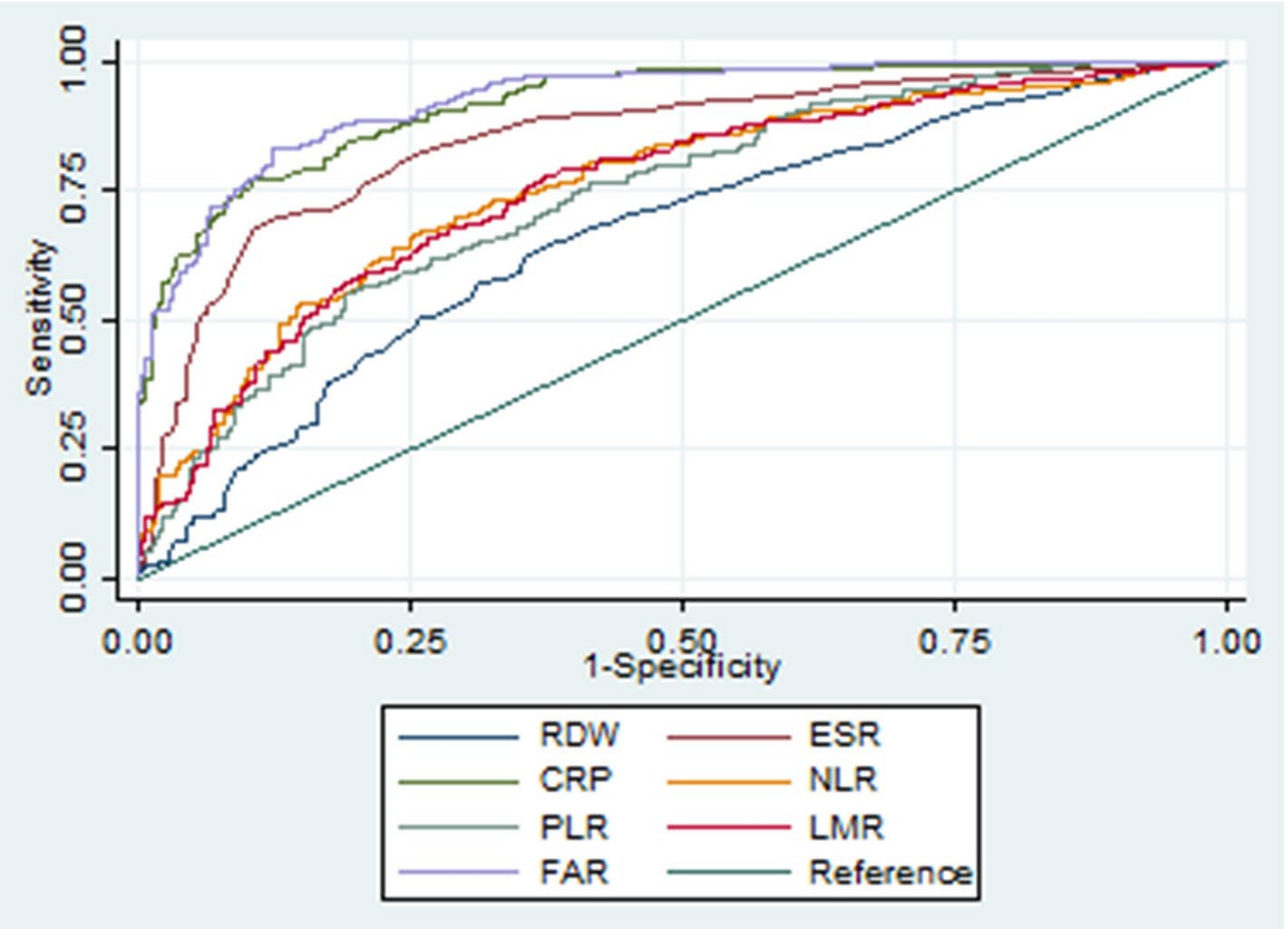
Receiver operating characteristic curves of various biomarkers in identifying active Crohn’s disease. RDW, red cell distribution width; ESR, erythrocyte sedimentation rate; CRP, C-reactive protein; NLR, neutrophil-to-lymphocyte ratio; PLR, platelet-to-lymphocyte ratio; LMR, lymphocyte-to-monocyte ratio; FAR, fibrinogen-to-albumin ratio.

Our study included 788 IBD patients, of whom 245 (31.1%) had UC and 543 (68.9%) had CD. Overall, patients in the UC group were older than those in the CD group. In addition, the CD group had a higher proportion of male patients than the UC group. More details about the characteristics of the IBD cohort are listed in Table 1. The FAR, CRP, ESR, PLR, and NLR levels in both active UC and CD patients scored significantly higher compared with those in the remission stage, whereas the LMR levels scored significantly lower (all *p* < 0.001), as shown in Tables 2, 3. Multivariate logistic regression analysis identified that FAR and CRP were independent predictors for identifying both active UC and CD. Table 4 lists the multivariate logistic regression analysis results. Table 5 presents significantly positive correlations of both Total Mayo Score and CDAI with FAR, NLR, PLR, RDW, CRP, and ESR, and negative correlations with LMR (all *p* < 0.001). Table 5 indicates correlations among biomarkers and disease activity of IBD. FAR, RDW, ESR, CRP, NLR, and PLR had significant positive correlations with the Total Mayo Score of UC, whereas LMR was negatively related to the Total Mayo Score of UC (all *p* < 0.001). In addition, the correlation analysis showed significant positive correlations of CDAI with the FAR, RDW, ESR, CRP, NLR, and PLR and negative correlations with LMR (all *p* < 0.001). Figures 1, 2 show that the FAR gave the highest area under the curve in identifying both active UC and CD compared with NLR, LMR, PLR, RDW, ESR, and CRP (all *p* < 0.05). Referring to the area under the curve (AUC) of these markers, we found that, while the FAR is better than CRP in identifying active CD, the difference is not significant. Tables 6, 7 show more details about the diagnostic performance of biomarkers. According to the FAR classification of UC patients (Group A: <0.076 and Group B: ≥0.076), the proportion of active UC patients in Groups A and B was 30.8% (24/78) and 88.6% (148/167), respectively, and the difference was statistically significant (*p* < 0.001). According to the FAR classification of CD patients (Group C: <0.107 and group D: ≥0.107), the incidence of active CD in Groups C and D was 18.2% (50/275) and 88.1% (236/268), respectively (*p* < 0.001). It can be seen that the probability of IBD patients being active increased significantly when the FAR was more than or equal to the optimal threshold. Based on the FAR classification for UC patients (Group A: <0.076 and Group B: ≥0.076), the active UC patients’ rates among Groups A and B were 30.8% (24/78) and 88.6% (148/167), respectively (*p* < 0.001). Based on the FAR classification for CD patients (Group C: <0.107 and Group D: ≥0.107), the active CD patients’ rates among Groups C and D were 18.2% (50/275) and 88.1% (236/268), respectively (*p* < 0.001). It is clear that the probability of IBD patients in the active stage significantly increased when the FAR was more than or equal to the optimal cutoff values.

**Table 1:** Characteristics of the inflammatory bowel disease cohort.

Parameter	UC cohort (*N* = 245)	CD cohort (*N* = 543)
Age (years)	49 (37–60)	27 (22–33)
Sex: male	129 (52.7)	396 (72.9)
BMI (kg/m^2^)	19.5 (18.4–21.2)	18.9 (17.4–20.9)
Smoking: yes	39 (15.9)	84 (15.5)
Drinking: yes	22 (9.0)	44 (8.1)
Duration of disease (years)	1.7 (0.5–4.0)	1.8 (0.6–3.5)
Endoscopic inflammatory localization of disease		
Proctitis	45 (18.4)	-
Left-side colitis	107 (43.7)	-
Extensive colitis	93 (38.0)	-
Terminal ileitis	-	130 (23.9)
Colitis	-	107 (19.7)
Ileocolitis	-	306 (56.4)
Remission stage	73 (29.8)	257 (47.3)
Active stage	172 (70.2)	286 (52.7)

Values are expressed as *n* (%) or median (IQR). UC, ulcerative colitis; CD, Crohn’s disease; BMI, body mass index.

**Table 2:** Epidemiology and laboratory parameters of ulcerative colitis patients, stratified by disease activity.

Parameter	UC remission (*N* = 73)	UC active (*N* = 172)	*p*-value
Age (years)	47 (37–57)	50 (38–61)	0.331
Sex: male	32 (43.8)	97 (56.4)	0.072
BMI (kg/m^2^)	20.6 (18.8–21.7)	19.3 (18.3–20.8)	0.001
Smoking: yes	10 (13.7)	29 (16.9)	0.536
Drinking: yes	7 (9.6)	15 (8.7)	0.828
Duration of disease (years)	2.0 (0.7–4.3)	1.1 (0.3–3.8)	0.042
Neutrophils (10^9^/L)	3.3 (2.7–4.4)	4.6 (3.4–7.2)	<0.001
Monocytes (10^9^/L)	0.4 (0.4–0.6)	0.7 (0.5–0.9)	<0.001
Lymphocytes (10^9^/L)	1.7 (1.5–2.2)	1.8 (1.3–2.2)	0.428
Hb (g/dL)	12.8 (12.0–13.5)	11.9 (10.4–13.2)	<0.001
RDW (%)	13.2 (12.7–13.9)	13.3 (12.7–14.3)	0.635
Platelet (10^9^/L)	235 (201–270)	281 (214–376)	<0.001
PT (seconds)	13.4 (12.9–13.7)	13.7 (13.1–14.6)	<0.001
INR	1.0 (1.0–1.1)	1.1 (1.0–1.1)	<0.001
Fibrinogen (g/L)	2.7 (2.3–3.2)	4.0 (3.0–5.0)	<0.001
CRP (mg/L)	3.0 (1.5–3.2)	12.8 (3.8–32.8)	<0.001
ESR (mm/h)	6 (2–15)	21 (11–37)	<0.001
Albumin (g/L)	41.6 (38.6–44.8)	33.6 (30.5–38.8)	<0.001
NLR	1.8 (1.3–2.7)	2.8 (1.9–4.2)	<0.001
PLR	130.0 (100.0–174.1)	166.4 (121.6–222.5)	<0.001
LMR	4.2 (3.4–5.1)	2.7 (1.8–3.5)	<0.001
FAR	0.063 (0.057–0.078)	0.116 (0.084–0.153)	<0.001

Values are expressed as *n* (%) or median (IQR), compared by the Mann–Whitney *U*-test and the chi-square test or Fisher’s exact test (*p* < 0.05 was considered statistically significant). UC, ulcerative colitis; BMI, body mass index; Hb, hemoglobin; RDW, red cell distribution width; PT, prothrombin time; INR, international normalized ratio; CRP, C-reactive protein; ESR, erythrocyte sedimentation rate; NLR, neutrophil-to-lymphocyte ratio; PLR, platelet-to-lymphocyte ratio; LMR, lymphocyte-to-monocyte ratio; FAR, fibrinogen-to-albumin ratio.

**Table 3:** Epidemiology and laboratory parameters of Crohn’s disease patients, stratified by disease activity.

Parameter	CD remission (*N* = 257)	CD active (*N* = 286)	*p*-value
Age (years)	27 (22–30)	26 (22–36)	0.641
Sex: male	185 (72.0)	211 (73.8)	0.639
BMI (kg/m^2^)	19.6 (18.3–21.6)	18.2 (16.4–20.2)	<0.001
Smoking: yes	35 (13.6)	49 (17.1)	0.258
Drinking: yes	14 (5.4)	30 (10.5)	0.032
Duration of disease (years)	2.0 (1.0–3.5)	1.1 (0.3–3.5)	<0.001
Neutrophils (10^9^/L)	3.5 (2.7–4.4)	5.1 (3.5–7.1)	<0.001
Monocytes (10^9^/L)	0.5 (0.4–0.6)	0.7 (0.5–0.9)	<0.001
Lymphocytes (10^9^/L)	1.5 (1.1–1.9)	1.2 (0.9–1.7)	<0.001
Hb (g/dL)	13.5 (12.2–14.7)	11.5 (10.1–12.6)	<0.001
RDW (%)	13.4 (12.7–14.9)	14.8 (13.3–16.6)	<0.001
Platelet (10^9^/L)	246 (206–294)	328 (254–414)	<0.001
PT (seconds)	13.5 (13.1–14.0)	14.0 (13.3–14.7)	<0.001
INR	1.1 (1.0–1.1)	1.1 (1.0–1.2)	<0.001
Fibrinogen (g/L)	3.0 (2.4–3.6)	4.7 (3.9–5.6)	<0.001
CRP (mg/L)	3.0 (1.6–8.6)	30.0 (15.6–60.4)	<0.001
ESR (mm/h)	7 (2–14)	31 (17–48)	<0.001
Albumin (g/L)	41.8 (38.2–45.1)	33.3 (30.2–36.0)	<0.001
NLR	2.2 (1.6–3.3)	4.1 (2.8–6.2)	<0.001
PLR	168.3 (118.8–231.9)	264.8 (192.1–370.0)	<0.001
LMR	3.0 (2.3–4.2)	1.9 (1.4–2.6)	<0.001
FAR	0.069 (0.057–0.092)	0.142 (0.117–0.177)	<0.001

Values are expressed as *n* (%) or median (IQR), compared by the Mann–Whitney *U*-test and the chi-square test or Fisher’s exact test (*p* < 0.05 was considered statistically significant). CD, Crohn’s disease; BMI, body mass index; Hb, hemoglobin; RDW, red cell distribution width; PT, prothrombin time; INR, international normalized ratio; CRP, C-reactive protein; ESR, erythrocyte sedimentation rate; NLR, neutrophil-to-lymphocyte ratio; PLR, platelet-to-lymphocyte ratio; LMR, lymphocyte-to-monocyte ratio; FAR, fibrinogen-to-albumin ratio.

**Table 4:** Multivariate logistic regression analysis results of disease activity for patients with inflammatory bowel disease.

		95% CI		
	OR	Lower	Upper	P-value
UC				
CRP	1.059	1.001	1.122	0.048
FAR	24.871	9.831	38.912	<0.001
CD				
Age	1.047	1.014	1.082	0.005
Sex: male	6.386	2.713	15.032	<0.001
BMI	0.755	0.666	0.856	<0.001
Duration of disease	0.988	0.979	0.997	0.011
Neutrophils	1.301	1.076	1.573	0.007
Lymphocytes	0.551	0.321	0.947	0.031
Hb	0.535	0.433	0.660	<0.001
CRP	1.084	1.042	1.128	<0.001
FAR	28.966	21.009	37.250	<0.001

ORs and *p*-values were estimated using multivariate logistic regression. Age, sex, and variables that were statistically significant in the tests were included in the multivariate analysis. OR, odds ratio; CI, confidence interval; UC, ulcerative colitis; BMI, body mass index; CRP, C-reactive protein; CD, Crohn’s disease; Hb, hemoglobin; FAR, fibrinogen-to-albumin ratio.

**Table 5:** Spearman’s correlation coefficients between biomarkers and disease activity of patients with inflammatory bowel disease.

	UC (Total Mayo Score)		CD (CDAI)	
Biomarkers	*r*-value	*p*-value	*r*-value	*p*-value
RDW	0.147	0.022	0.361	<0.001
ESR	0.438	<0.001	0.636	<0.001
CRP	0.599	<0.001	0.753	<0.001
NLR	0.399	<0.001	0.444	<0.001
PLR	0.311	<0.001	0.428	<0.001
LMR	−0.499	<0.001	−0.452	<0.001
FAR	0.676	<0.001	0.758	<0.001

*p- and r-values were estimated using Spearman’s correlation analysis. UC, ulcerative colitis; CD, Crohn’s disease; CDAI, Crohn’s disease activity index; RDW, red cell distribution width; ESR, erythrocyte sedimentation rate; CRP, C-reactive protein; NLR, neutrophil-to-lymphocyte ratio; PLR, platelet-to-lymphocyte ratio; LMR, lymphocyte-to-monocyte ratio; FAR, fibrinogen-to-albumin ratio.*

**Table 6:** Diagnostic accuracy of various biomarkers in identifying ulcerative colitis in the active stage.

Biomarkers	AUROC	95% CI	*p*-value	Cut-off	Sensitivity	Specificity	PV+	PV−	LR+	LR−
RDW	0.519	0.443–0.596	<0.001	13.7	0.40	0.71	0.77	0.34	1.39	0.84
ESR	0.764	0.701–0.828	0.001	16.0	0.67	0.77	0.87	0.50	2.90	0.42
CRP	0.814	0.760–0.868	0.040	3.32	0.79	0.75	0.88	0.60	3.21	0.28
NLR	0.683	0.611–0.756	<0.001	2.01	0.67	0.63	0.81	0.45	1.82	0.52
PLR	0.643	0.570–0.716	<0.001	132	0.70	0.56	0.79	0.44	1.59	0.54
LMR	0.762	0.697–0.826	0.004	3.59	0.77	0.73	0.87	0.58	2.82	0.31
FAR	0.870	0.824–0.916	Ref	0.076	0.86	0.74	0.89	0.69	3.31	0.19

DeLong test was used to compare the AUROC between fibrinogen-to-albumin ratio and other biomarkers and estimate *p*-values and FAR was the reference. AUROC, area under the receiver operating characteristic curve; CI, confidence interval; PV+, positive predictive value; PV−, negative predictive value; LR+, positive likelihood ratio; LR−, negative likelihood ratio; RDW, red cell distribution width; ESR, erythrocyte sedimentation rate; CRP, C-reactive protein; NLR, neutrophil-to-lymphocyte ratio; PLR, platelet-to-lymphocyte ratio; LMR, lymphocyte-to-monocyte ratio; FAR, fibrinogen-to-albumin ratio.

**Table 7:** Diagnostic accuracy of various biomarkers in identifying Crohn’s disease in the active stage.

Biomarkers	AUROC	95% CI	*p*-value	Cutoff	Sensitivity	Specificity	PV+	PV−	LR+	LR−
RDW	0.661	0.615–0.707	<0.001	14.0	0.65	0.62	0.66	0.61	1.71	0.57
ESR	0.849	0.816–0.882	<0.001	21.0	0.70	0.87	0.86	0.72	5.45	0.35
CRP	0.916	0.893–0.938	0.374	14.8	0.77	0.89	0.89	0.78	7.32	0.26
NLR	0.758	0.717–0.798	<0.001	3.32	0.66	0.75	0.75	0.67	2.65	0.45
PLR	0.739	0.698–0.781	<0.001	247	0.57	0.79	0.75	0.62	2.75	0.55
LMR	0.754	0.714–0.795	<0.001	2.67	0.79	0.61	0.69	0.73	2.04	0.34
FAR	0.925	0.904–0.946	Ref	0.107	0.83	0.88	0.88	0.82	6.66	0.20

DeLong test was used to compare the AUROC between fibrinogen-to-albumin ratio and other biomarkers and estimate *p*-values and FAR was the reference. AUROC, area under the receiver operating characteristic curve; CI, confidence interval; PV+, positive predictive value; PV−, negative predictive value; LR+, positive likelihood ratio; LR−, negative likelihood ratio; RDW, red cell distribution width; ESR, erythrocyte sedimentation rate; CRP, C-reactive protein; NLR, neutrophil-to-lymphocyte ratio; PLR, platelet-to-lymphocyte ratio; LMR, lymphocyte-to-monocyte ratio; FAR, fibrinogen-to-albumin ratio.

## Discussion

The research externally validated for the first time a new and non-invasive biomarker, the FAR’s diagnostic ability to identify active IBD patients.

Timely diagnosis of the disease activity of IBD can help us choose the best treatment plan and improve the prognosis of patients. Endoscopic biopsy remains the gold standard for assessing and monitoring inflammatory activity in IBD. Fecal calprotectin was a good biological index to evaluate IBD activity (27–29). However, this index had a high cost, long time, and inconvenient sample collection and processing. Thus, there is an urgent need for a simple, readily obtainable, low-cost, and efficient biomarker to optimally manage IBD patients.

Fibrinogen deposits are a near-universal feature of tissue injury, including injury driven by immunological derangements (19). Low albumin levels often indicate malnutrition, while patients’ nutriture is associated with disease activity, and the nutriture of active IBD patients is worse. In addition, the inflammatory response can influence albumin synthesis (30). Thus, we proposed the FAR to identify active IBD patients. The FAR has its unique advantages. First, the FAR can be rapidly calculated using two parameters and an easy formula without the need for a tedious calculation process. Second, fibrinogen and albumin levels can be easily determined using an inexpensive and non-invasive blood test. The FAR is a biomarker of high prediction accuracy and practicality and is objectively assessed. Third, the FAR is a potential biomarker that may have high accuracy and practicality. According to our findings, a FAR greater than 0.076 in UC and 0.107 in CD may indicate the active phase of the disease. Of course, the value of FAR requires further verification by a prospective multi-center large-sample study. Finally, the FAR may have potential clinical applications in the evaluation of the therapeutic effect and prediction of the complications of IBD. These need further study.

However, the research has its limitations. First, this study was retrospective single-center research. We will conduct a prospective multi-center large-sample study in the future. In addition, we did not take into account several factors, including immunosuppressant and corticosteroid use, which may have an impact on inflammatory biomarker levels. In the following research, we will distinguish between new cases and those that are under treatment. Finally, we included some commonly used inflammatory biomarkers but others were excluded. Problems will be addressed in the following studies.

In conclusion, the laboratory-based FAR is a simple and practical biomarker to identify active IBD patients. Further study is needed to explore and validate other potential clinical application values of FAR.

## Data Availability

The raw data supporting the conclusions of this article will be made available by the authors, without undue reservation.
